# Automated Identification, Warning, and Visualization of Vortex-Induced Vibration

**DOI:** 10.3390/s25196169

**Published:** 2025-10-05

**Authors:** Min He, Peng Liang, Xing-Shun Lu, Yu-Hao Pan, Di Zhang

**Affiliations:** 1 School of Architecture and Civil Engineering, Xi’an University of Science and Technology, Xi’an 710054, China; hemin0214@163.com; 2 Research Center of Highway Large Structure Engineering on Safety, Ministry of Education, Chang’an University, Xi’an 710064, China; lxspen2001@163.com (X.-S.L.); 18209272741@163.com (Y.-H.P.); zhangdi@schdri.com (D.Z.); 3 Highway School, Chang’an University, Xi’an 710064, China

**Keywords:** vortex-induced vibration, recurrence plot, feature index, automatic identification, multi-level warning, visualization

## Abstract

Vortex-induced vibration (VIV) is a kind of abnormal vibration which needs to be automatically identified and warned in real time to guarantee the operational safety of a bridge. However, the existing VIV identification methods only focus on identification and have limitations in visualizing identification results, which causes difficulty for bridge governors in other fields to quickly confirm the identification results. This paper proposes an automatic VIV identification, warning, and visualization method. First, a recurrence plot is introduced to analyze the signal to extract the characteristics of the vibration signal in a time domain. Then, a feature index defined as recurrence cycle smoothness is proposed to quantify the stability of the vibration signal, based on which the VIV can be automatically identified. An automatic VIV identification and multi-level warning process is finally established based on the severity of the vibration amplitude. The proposed method is validated through a suspension bridge with serious VIVs. The result indicates that the proposed method can automatically identify the VIV correctly without any manual intervention and can visualize the identification results using a graph, providing a good tool to quickly confirm the VIV identification results. The multi-level warning can successfully warn the serious VIV and provide possible early warning for large amplitude VIV.

## 1. Introduction

Slender structures such as bridges and stay cables in wind environments are always susceptible to wind-induced vibration due to high flexibility and low damping [1,2,3]. The vortex-induced vibration (VIV) is an abnormal wind-induced vibration which might occur in long-span bridges [4,5,6]. Serious VIV would cause large vibration amplitude of the girder and lead to a driving risk for the drivers on the bridge and even cause social panic. Many VIVs in bridges have been observed and reported and have caused much social impact [7], which brings much pressure for bridge governors. Therefore, motoring and identifying VIV is always needed and an important task in the practical monitoring of bridges.

Before identifying VIV, knowing the mechanical principle of VIV is the priority goal. Many researchers investigated VIV using CFD and wind tunnel experiments. Diana [8], Li [9], Ma [10], Hwang [3], Li [11], Li [12], Laima [13] et al. conducted wind tunnel experiments to investigate the phenomenon of vortex shedding, the effects of Reynolds number, and the effect of ancillary structures to aerodynamic characteristics. These studies aim to predict structural performance under VIV and evaluate the effect of control measures to VIV. These studies are mainly conducted during the design phase and focused on examining the mechanism of VIV and testing the vibration suppression methods to assess design rationality.

Some researchers installed some accelerometers on the bridges and cables to monitor the vibration response during VIV and study the mechanism between the structure and wind. These studies mainly focus on the correlation of VIV with environmental factors. Fujino et al. [14] examined the correlation between VIV and wind speed and direction based on monitoring data and pointed out that VIV is highly correlated with wind speed and direction. Ge et al. [15] investigated the spectral characteristics and VIV amplitudes based on half-year monitoring data from the stay cables of the Sutong Bridge and explored the correlation of VIV with environmental factors. The results indicate that VIV only occurs in specific wind speeds and wind directions. Chen et al. [16] studied the relationship between wind and VIV based on two months of monitoring data, and confirmed that VIV occurs under low wind speeds and specific wind directions. Yang [17] conducted a full-scale measurement on the structural dynamic characteristics and vortex-induced vibrations (VIV) of a long-span suspension bridge with a central span of 1650 m, and compared the VIV response between finite element model and real monitoring. These studies consistently concluded the same phenomena, such as the wind speed lock-in phenomenon for VIV and its strong dependence on wind direction. Through qualitative descriptions, the existing studies systematically elucidated the feature characteristics of VIV signals in time domain and frequency domain, which establishes a foundational understanding for researchers to establish the VIV identification method.

With the development of structural health monitoring, many structural health monitoring systems have been established, and many sensors are installed permanently on the bridge to monitor the vibration response. Based on the achievements in theoretical and experimental studies, many VIV identification methods were proposed. Most of the methods rely on proposing a feature index to distinguish VIV from ambient vibration. Li et al. [18] proposed an automated VIV identification method using cluster analysis based on four years of monitoring data from a suspension bridge girder. This approach employs the energy concentration coefficient and root mean square of acceleration derived from frequency-domain and time-domain features of VIV signals as characteristic indices. Xu et al. [19] analyzed the correlation between wind parameters and acceleration RMS and established a VIV identification method which uses wind speed and wind direction as preconditions and energy concentration coefficient and acceleration RMS as feature indices. However, this method requires a manual threshold definition, and thresholds need to be adjusted with different bridges. Li et al. [20] developed a data-driven model for VIV response prediction using decision trees and support vector regression to model VIV responses from nearly six years of field monitoring from a suspension bridge. Huang et al. [21] utilized the random decrement technique to analyze the vibration signals and utilized the variation coefficient to identify VIV. Dan et al. [22] employed recursive Hilbert transform to calculate the vibration displacement of the girder and identified VIV using a displacement-based threshold. The main contribution of the method is that it can reconstruct the vibration displacement of VIV. He et al. [23,24] proposed two feature indices from frequency domain and complex domain to describe the characteristics of the VIV signal in frequency domain and time domain. These two features were then used to construct a feature vector in the process of VIV identification. The k-means clustering method was utilized to automatically separate the vibration states into VIV and ambient vibration. With the development of artificial intelligence (AI), some AI-based methods were proposed to fulfill the automatic VIV identification. Kim et al. [25] developed a general framework for introducing ML algorithms to predict VIVs with a limited amount of information. In this method, a variety of ML-assisted methods are introduced to predict VIVs. The effectiveness and applicability of the proposed framework are demonstrated using actual monitoring data. Arul et al. [26] proposed an unsupervised, robust machine-learning technique to identify the VIV of a building and evaluated the fatigue life of a tall building. Su et al. [27] proposed a novel framework using the multimodal fusion techniques with deep neural networks to autonomously identify the VIV suspenders. In this method, two vibration response parameters are identified as indexes for the identification of suspenders VIV based on the significant difference method of big data analysis, and the convolutional neural network and bidirectional long short-term memory are utilized to extract the suspenders vibration response features. The method was proven to be effective based on the application to suspenders. Yang et al. [28] established a prediction model to predict the amplitude of the double-deck steel truss girder under VIV based on three machine learning algorithms, and the results indicate that the established model can be used for VIV response prediction and is more efficient compared to the numerical simulation method.

In real application, the VIV identification methods are expected to integrate an online monitoring software to fulfill the online automatic VIV identification and issue distinct visualized results for engineers with different backgrounds to quickly obtain and confirm the identification result. In most cases, the governors of bridges do not have much knowledge of bridges or VIVs, and a distinct visualized identification result is always required for bridge governors to quickly and intuitively confirm the VIV identification results. Even though many methods have been proposed to automatically identify the VIV, the existing methods mainly focus on identification and have limits in results visualization. Therefore, a VIV identification method with good ability both in identification and visualization is required.

This paper aims to establish an automatic VIV identification method with good ability in identification and visualization. The recurrence plot is introduced as the basic tool to process the signal and visualize the identification results. A new index is proposed to identify the VIV and a multi-level warning system is established for early warning. The main contributions of this study include the following:

(1) Introducing an automated signal processing method to extract the characteristics of the vibration signal.
(2) Proposing a feature index to quantify the stability of the vibration signal in time domain for VIV identification.
(3) Establishing a multi-level warning strategy based on acceleration for VIV warning.
(4) Producing visualized VIV identification results for quick confirmation.

The following of the paper is organized as follows: Section 2 describes the mechanical principle of VIV, and the feature characteristics which can be used for VIV identification are also summarized in this section. Section 3 introduces the proposed VIV identification and warning methods. A real application of the proposed method to a suspension bridge is presented in Section 4, and conclusions are summarized in Section 5.

## 2. Description of VIV

A vortex-induced vibration is a wind-induced resonance phenomenon that occurs in slender structures, such as long-span bridges under low wind speeds. As wind flows past a slender structure, the vortex shedding forms up behind the structure and causes the structure to vibrate under the vortex-induced forces. The VIV occurs when the frequency of the periodic vortex shedding is close to the natural frequency of the structure. The VIV can be qualitatively interpreted using some mechanical models [29] where the vibration of the structure can be described as

(1) $$m\left(\ddot{y}+2\xi \omega \dot{y}+\omega^{2}y\right)=F_{VI}$$

where y is the vertical displacement of the deck sectional model; *m* denotes the mass per unit length; ω is the structural circular frequency; ξ=c2mω1 is the mechanical damping ratio and *c* is the structural damping; $F_{VI}$ is the vortex-induced force and can be modeled using sinusoidal load.

Based on the model, the VIV displacement *y* of the structure can be approximately expressed as a narrow-band harmonic model as:

(2) y(t)=a(t)sin(ωt+ϕ)+noise

where *t* is the time; ω is the dominant vibration frequency of structure during VIV; ϕ is the phase; and a(t) is the amplitude of VIV. The theoretical analysis proves that single-mode vibration is the characteristic of VIV, and the vibration signal of VIV is harmonic, which differs the VIV from the ambient vibration.

**Figure 1:**
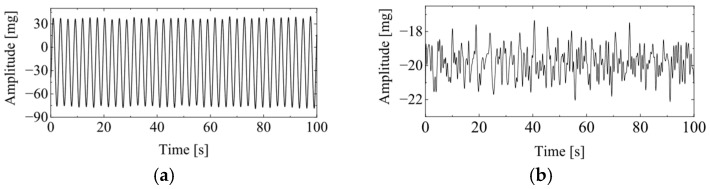
Typical vibration signals for VIV and ambient vibration. (**a**) VIV signal; (**b**) ambient vibration signal.

Based on this characteristic, it can be concluded that VIV is a single-mode vibration with stable amplitude, and two characteristics of VIV can be summarized as follows: (1) VIV is single mode vibration and (2) the amplitude of VIV is stable. The typical vibration signal for VIV and ambient vibration is presented in Figure 1. The main work for VIV identification is to automatically distinguish the vibration signal of VIV from that of the ambient vibration.

Equations (1) and (2) are a qualitative description of VIV, and it is suitable for any structure such as bridges and stay cables. Any structures having VIV would have the same vibration signal characteristics described above. In this study, the proposed method would focus on analyzing the vibration signal, and any structure having VIV signal would be identified.

## 3. Proposed Automatic Identification and Warning Method

### 3.1. Recurrence Plot

The recurrence plot (RP) is a signal process method used to visualize the results of phase space reconstruction in dynamical systems [30]. This technique maps the trajectory in phase space into a two-dimensional matrix, where each element of the matrix represents the distance between two trajectory points in phase space. In the recurrence plot, if the distance between two trajectory points is less than a specified threshold, the corresponding matrix element is marked as 1; otherwise, it is marked as 0. Phase space reconstruction provides the input data for recurrence plots, which helps to analyze the characteristics of dynamical systems by visualizing the reconstructed phase space.

The recurrence plots map time series into phase space to detect the repetition (recurrence) of system states over time by firstly carrying out the phase space reconstruction as

(3) $$x\left(i\right)=\left\{x\left(i\right),x\left(i+\tau \right),\ldots ,x\left(i+\left(m-1\right)\tau \right)\right\}$$

where $x\left(i\right)$ denotes the phase space vector; $x\left(i+\left(m-1\right)\tau \right)$ denotes the $\left(i+\left(m-1\right)\tau \right)\mathrm{th}$ element of the *m*-dimensional time series signal $S\left(i\right)$; *m* denotes embedding dimension; τ denotes time delay.

Based on the phase space reconstruction, the recursive matrix can be established by

(4) R(i,j)=Θ(ϵ−‖x(i)−x(j)‖)

where R(i,j) is a $i+\left(m-1\right)\tau$ dimensional matrix; Θ denotes Heaviside function, and Θ is 1 when ε>‖x(i)−x(j)‖, while it is 0 when ε<‖x(i)−x(j)‖; ‖ ‖ denotes the Euclidean norm; ε denotes the recurrence threshold and is normally set as 0.1–0.3. The recurrence plot is a graphic display of the recursive matrix where 1 is plotted as solid circle and 0 is plotted as hollow circle. By observing the distribution of the solid circles, the characteristic of the signal can be analyzed.

### 3.2. VIV Identification Method

#### 3.2.1. Determination of Time Delay

In phase space reconstruction, the time delay τ is a critical parameter that ensures a reconstructed phase space vector to maintain correlation to preserve system dynamical information. The goal of the time delay is to determine the optimal *τ* that maximizes the preservation of the original system’s dynamics in the reconstructed phase space. The Mutual Information (MI) method [31] is utilized to determine *τ*. The core principle of MI method measures the degree of dependence between different temporal points according to ‘mutual information’ from information theory and identifies the delay when mutual information first drops to a characteristic value.

Suppose two discrete sequences $S=\left\{s_{1},s_{2},\cdots ,s_{n}\right\}$ and $Q=\left\{q_{1},q_{2},\cdots ,q_{n}\right\}$ represent systems S and Q, respectively. Based on the information theory, the information entropy of the two systems can be estimated by

(5) $$H(S)=-\sum_{i=1}^{n}P_{S}(s_{i})\log_{2}P_{S}(s_{i})$$

(6) $$H(Q)=-\sum_{j=1}^{n}P_{Q}(q_{j})\log_{2}P_{Q}(q_{j})$$

where $P_{S}\left(s_{i}\right)$ and $P_{Q}\left(q_{j}\right)$ denote the probabilities of events $s_{i}$ and $q_{j}$.

The mutual information between *Q* and *S* can be calculated by

(7) I(S,Q)=H(S)−H(S|Q)

Then $H(S|q_{j})$ can be calculated as

(8) $$H(S|q_{j})=-\sum_{i}\left[P_{QS}(q_{j},s_{i})/P_{Q}(q_{j})]\log[P_{QS}(q_{j},s_{i})/P_{Q}(q_{j})\right]$$

Then $I\left(S,Q\right)$ can be written as

(9) $$I(S,Q)=\sum_{j}\sum_{i}P_{QS}(q_{j},s_{i})\log_{2}[\frac{P_{QS}(q_{j},s_{i})}{P_{Q}(q_{j})P_{S}(s_{i})}]$$

where $P_{QS}\left(q_{j},s_{i}\right)$ denotes the joint distribution probability of events $q_{j}$ and $s_{i}$.

In the above definition, if we suppose *Q* represents the time series $x\left(t\right)$ and *S* represents $x\left(t+\tau \right)$, then $I\left(S,Q\right)$ would be a function with τ, which is written as

(10) $$I\left(S,Q\right)=I\left(\tau \right)$$

The way of finding the optimal τ is to find the τ with the minimum $I\left(\tau \right)$, in which it would mean the corresponding two-phase space vectors are with the highest independence.

**Figure 2:**
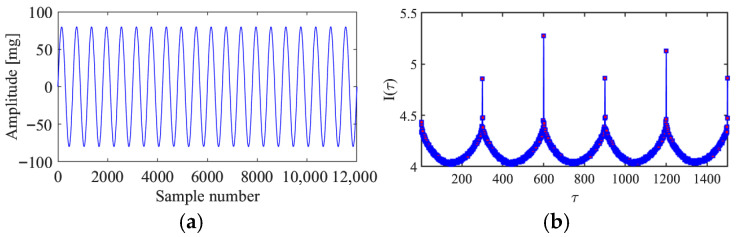
The VIV signal and determination of optimal time delay. (**a**) The VIV signal; (**b**) the evolution of $I\left(\tau \right)$ with time delay.

**Figure 3:**
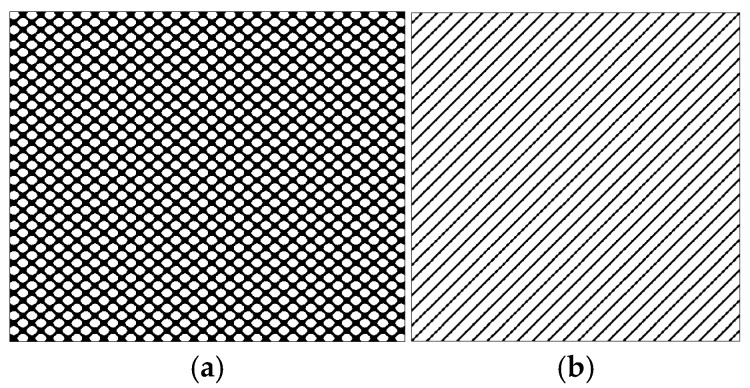
The recurrence plot of VIV signal with and without optimal time delay. (**a**) Without optimal time delay; (**b**) with optimal time delay.

According to the theory of time delay, it is easy to conclude that the optimal time delay for VIV signal, i.e., the sine wave signal, is one fourth of the natural period. Figure 2 presents the evolution of $I\left(\tau \right)$ with time delay for a period of VIV signal where the natural period is 60 s and the sampling frequency is 1 Hz. It can be seen that $I\left(\tau \right)$ changes periodically with time delay for VIV signal. The time delay corresponding to the first smallest $I\left(\tau \right)$ of VIV signal, which is 150, is selected as the optimal time delay. Figure 3 presents the recurrence plots of the VIV signal when the time delay is set as 1 (which means no time delay) and 150. It can be seen that the recurrence plot with the optimal delay time exhibits more pronounced periodic characteristics (diagonal line features) and is not affected by interference from lines perpendicular to the diagonal.

**Figure 4:**
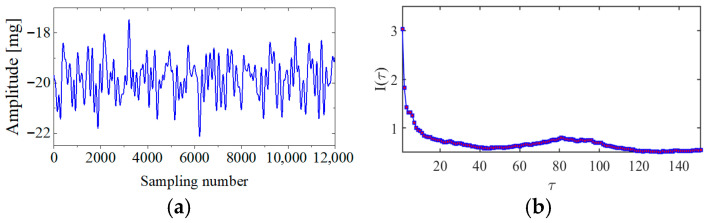
The ambient vibration signal and determination of optimal time delay. (**a**) The ambient vibration signal; (**b**) the evolution of $I\left(\tau \right)$ with time delay.

**Figure 5:**
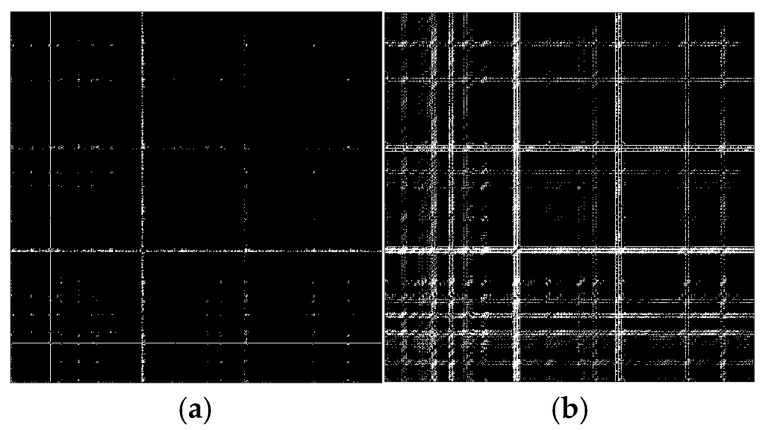
The recurrence plot of ambient vibration signal with and without optimal time delay. (**a**) Without optimal time delay; (**b**) with optimal time delay.

Figure 4 presents the evolution of $I\left(\tau \right)$ with time delay for a period of ambient vibration signal. Different with VIV signal, the $I\left(\tau \right)$ of ambient vibration signal decreases to a small value with no recycle pattern. Figure 5 presents the recurrence plots of the ambient vibration signal when the time delay is set as 1 and 43. In contrast, Figure 3 shows the recurrence plot of ambient vibration signal and displays no pattern even when the optimal time delay is set.

Figure 3 and Figure 5 show the differences in recurrence plot of VIV and ambient vibration signals. The recurrence plot shows multiple parallel diagonals for the VIV signal when optimal time delay is set, while it shows no pattern for ambient vibration even when the optimal time delay is set. The parallel diagonals reflect the stability of the VIV signal in time domain, and VIV and ambient vibration can be distinguished by identifying the pattern of recurrence plot. In the process of automatic VIV identification, the FNN method is firstly conducted for the segment of signal to calculate $I\left(\tau \right)$. Then the τ corresponding to the smallest $I\left(\tau \right)$ is automatically defined as the optimal time delay for signal processing.

#### 3.2.2. Determination of Embedding Dimension

The embedding dimension is another key parameter that needs to be carefully defined. In this study, the embedding dimension *m* is determined using the False Nearest Neighbors (FNN) method [32]. In the m-dimensional phase space, every phase space vector $x_{m}\left(i\right)$ has a nearest neighbor $x_{m}^{N}\left(i\right)$ within a certain distance, which can be written as

(11) $$d_{m}=\|x_{m}\left(i\right)-x_{m}^{N}(i)\|$$

Similarly, the smallest distance with a $x\left(i\right)$ in (m + 1)-dimensional phase space can be written as

(12) $$d_{m+1}=\|x_{m+1}\left(i\right)-x_{m+1}^{N}(i)\|$$

If the two points are false neighbors, the smallest distance will increase significantly when the dimensionality increases from *m* to *m* + 1, and the difference in the two smallest distances can be estimated by

(13) $$t=\frac{d_{m+1}}{d_{m}}$$

where t denotes the relative ratio.

True neighbors exhibit smaller distance variations in higher-dimensional spaces as they reflect the system’s intrinsic dynamical relationships, whereas false neighbors experience a sharp increase in distance due to the ‘completion’ of missing dimensions. Therefore, if *t* is bigger than a threshold, it means the corresponding two-phase space vectors are false neighbors, otherwise they are true neighbors. In practical applications, the threshold of *t* is normally set as 10%.

**Figure 6:**
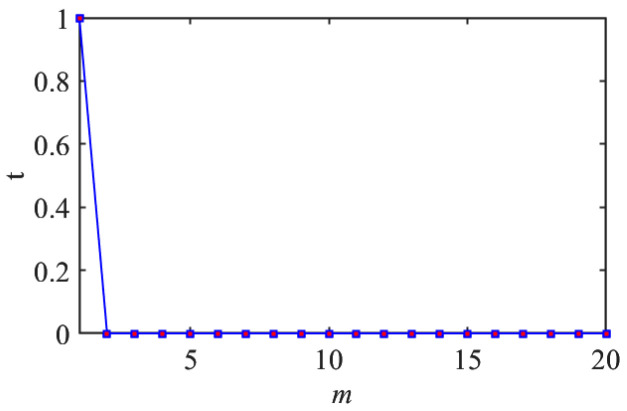
Determination of optimal embedding dimension.

**Figure 7:**
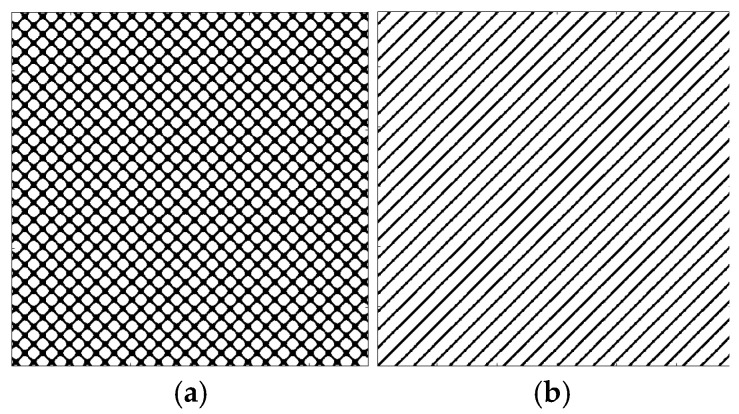
The recurrence plot of VIV signal with and without optimal embedding dimension. (**a**) Without optimal embedding dimension; (**b**) with optimal embedding dimension.

The embedding dimension is determined using the FNN method. Figure 6 presents the evolution of *t* with embedding dimensions. It can be seen that *t* decreases to 0 when the embedding dimension is 2, which means the smallest distances would not change when the embedding dimension is bigger than 2. Figure 7 presents the recurrence plots of the VIV signal when the embedding dimensions are set as 1 and 2, with time delay set as 150. The plots validate that the embedding dimension would influence the pattern of the recurrence plots and therefore would influence the VIV identification. In the process of identifying the VIV, the optimal embedding dimension is selected as the *m*, corresponding to the smallest *t*.

#### 3.2.3. Feature Index for Characterization of VIV and Identification

**Figure 8:**
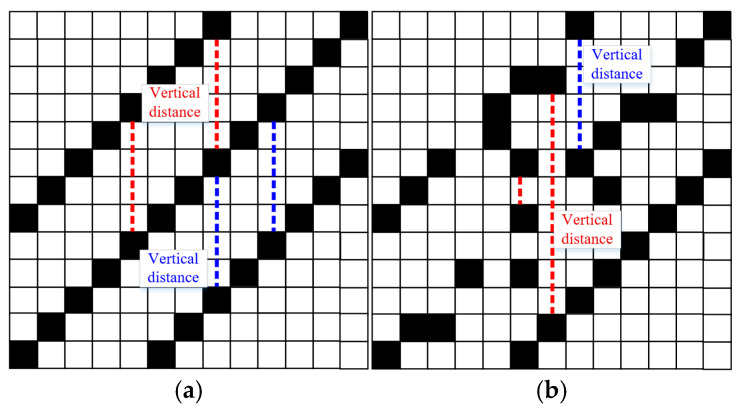
Explanation of vertical distance. (**a**) For VIV signal; (**b**) for ambient vibration signal.

The recurrence plot visualizes the characteristic of the signal and can distinguish the VIV and the ambient vibration visually. However, in real applications, the VIV identification needs to be conducted automatically for early warning. To reach this goal, a feature index which can describe the characteristics of the recurrence plots of the VIV signal and ambient vibration signal is proposed. For a VIV signal with the optimal time delay and embedding dimension, the recurrence plot would show multiple parallel diagonal lines and the vertical distance between these diagonal lines would be stable. For an ambient vibration signal, no specific pattern can be observed and the vertical distance would have significant discreteness. The vertical distance of the VIV signal and ambient vibration signal can be explained in Figure 8. Based on this characteristic, a feature index called recurrence cycle smoothness (RCS) is proposed for VIV identification and defined as

(14) $$\mathrm{RCS}=\frac{\mathrm{mean}({\left(i_{m}-i_{n}\right)}_{j})}{\max({\left(i_{m}-i_{n}\right)}_{j})}\;j=1,2,\cdots ,N;\;m\leq N$$

where $i_{m}$ and $i_{n}$ denote the row locations of the adjacent two elements with a value of 1 in the *j*-th column. For the VIV signal, the vertical distance would be stable and the RCS would be close to 1, while it would be close to 0 for ambient vibration.

RCS estimates the stability of the signal in the time domain. In contrast to the existing indices, such as root mean square (RMS) and energy concentration coefficient, RCS can capture the characteristics of regular steady-state VIV signals and avoid the risks in VIV identification. Low vibration amplitude VIV would be missed using RMS and ambient vibration would be wrongly identified merely using the energy concentration coefficient.

**Figure 9:**
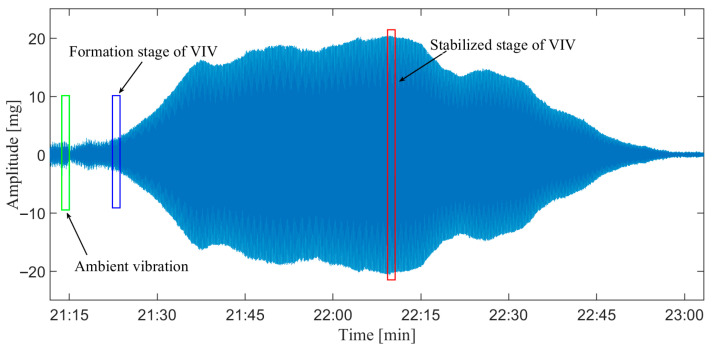
Real signal containing different stages of vibrations.

A segment of signal containing VIV and ambient vibration is utilized to validate the feasibility of the index and define a reasonable threshold for VIV identification. Figure 9 presents the vibration signal, and three segments of the signal corresponding to different vibration states are selected to analyze the RCS.

Table 1 presents the original signals and the corresponding recurrence plot, statistic of vertical distance, and RCS. It can be seen from Table 1 that the recurrence plots show different patterns for different signals corresponding to different vibration states. The recurrence plot of the stabilized VIV shows clear diagonal lines, which is obviously different from that for the ambient vibration. The recurrence plot of the signal for the formation stage of VIV also shows the characteristics of diagonal lines, but the edges of the lines are not clearer than those of the VIV signal.

The differences of the recurrence plots are also reflected in vertical distances and RCS values. It can be seen from the statistics of the vertical distance that most of the vertical distances of the recurrence plot for ambient vibration are close to 0, while most of the values are close to the maximum value for VIV signal. For this segment of VIV signal, the RCS is 0.895, which is much bigger than 0.023 for the ambient vibration signal, validating the feasibility of the proposed index in VIV identification. The vertical distances and RCS value of the VIV formation stage are between the values of the ambient vibration and VIV. These values validate that the characteristics of different vibration states can be reflected in RCS values, and different vibration states can be identified when reasonable thresholds are defined.

The RCS is a normalized index. For a VIV signal, it would be close to 1, while it would be close to 0 for ambient vibration. In real vibrations, the VIV amplitudes would still fluctuate in a small range due to the influence of environmental noises, which would result in the RCS of the VIV signal not being 1. The statistical results in Table 1 reveal the influence of the noise. It can be seen that even for the stable VIV signal, the RCS is not 1.0. A bigger threshold would lead to strict identification results, i.e., less VIV would be identified when a big threshold is set. In real applications, the thresholds can be defined based on the will of the engineers. In this study, to adapt to this practical condition, the thresholds for multi-stage VIV identification are defined as shown in Table 2.

The multi-stage identification aims to establish an early warning strategy that can identify the VIV in early stage. The ‘Ambient Vibration’ stage means the vibration is ambient. The ‘Incipient Stage’ means the vortex shedding is forming up, but the vibration is not stable. In this stage, the vibration might go to VIV or die as ambient vibration. The ‘Formation Stage’ means stable vortex shedding is happening, and the vibration most probably would go to stable VIV. The ‘Fully Developed Stage’ means the stable VIV is happening, and this is the most serious stage for VIV identification and warning.

#### 3.2.4. Multi-Level VIV Warning

In some cases, VIVs are with such small vibration amplitudes that the drivers and the people on the bridge cannot feel them. These VIVs should be identified, yet it is not necessary to issue an alarm. In real applications, an automatic warning strategy should be established based on the influence of severity on the people or the structure. Some standards also provide some instructions for VIV. AASHTO and “Guide Specifications for Wind Effects on Bridges” provide some instructions in the design process, such as adding the damping to mitigate the vibration. In “Guide Specifications for Wind Effects on Bridges”, it provides some restrictions in vertical and horizontal accelerations to guarantee the comfort of walkers on the bridge. Eurocode provides some restrictions for wind-induced vibration. For VIV, the code provides methods for estimating the maximum amplitude and requires that it should be controlled within an acceptable range. The Chinese “Highway and Bridge Wind-Resistance Design Standard JTG/T 3360-01—2018” clearly defines that the severity of VIV should be estimated according to the amplitude of the vibration displacement and stipulates that the allowable displacement amplitude for VIV can be determined as

(15) $$d_{i}^{a}=\frac{0.04}{f_{i}}$$

where $d_{i}^{a}$ denotes the allowable amplitude for the *i*-th mode VIV; $f_{i}$ denotes the dominant frequency of the VIV.

In actual bridge monitoring, the acceleration is monitored in all cases. Therefore, the allowable displacement amplitude needs to be transformed into allowable acceleration amplitude. When VIV occurs, the vibration signal is the sine wave signal, and the relationship between displacement and acceleration can be written as

(16) $$A_{i}=d_{i}\omega_{i}^{2}$$

where $A_{i}$ denotes the acceleration for the *i*-th mode VIV and $\omega_{i}$ denotes the dominant frequency corresponding to the *i*-th mode VIV. Then the allowable acceleration amplitude can be determined as

(17) $$A_{i}^{a}=\frac{0.04}{f_{i}}(4\pi^{2}f_{i}^{2})=1.58f_{i}$$

To quantify the severity of the vibration, a dimensionless coefficient is proposed as

(18) $$r_{i}=\frac{A_{i}^{m}}{A_{i}^{a}}$$

where $r_{i}$ demotes the severity ratio of the *i*-th mode VIV; $A_{i}^{m}$ denotes the monitored acceleration amplitude of the *i*-th mode VIV. When $r_{i}$ is smaller than 1, it means the acceleration amplitude is smaller than the allowable acceleration amplitude and no alarm needs to be issued. While when $r_{i}$ exceeds 1, it means the acceleration amplitude is bigger than the allowable acceleration amplitude and an alarm should be issued.

By computing the severity ratio, the influence of the VIV to the traffic and people on the bridge can be estimated. Thus, a multi-level warning strategy based on the severity ratio is established, as shown in Table 3.

#### 3.2.5. Automatic VIV Identification and Warning Process

**Figure 10:**
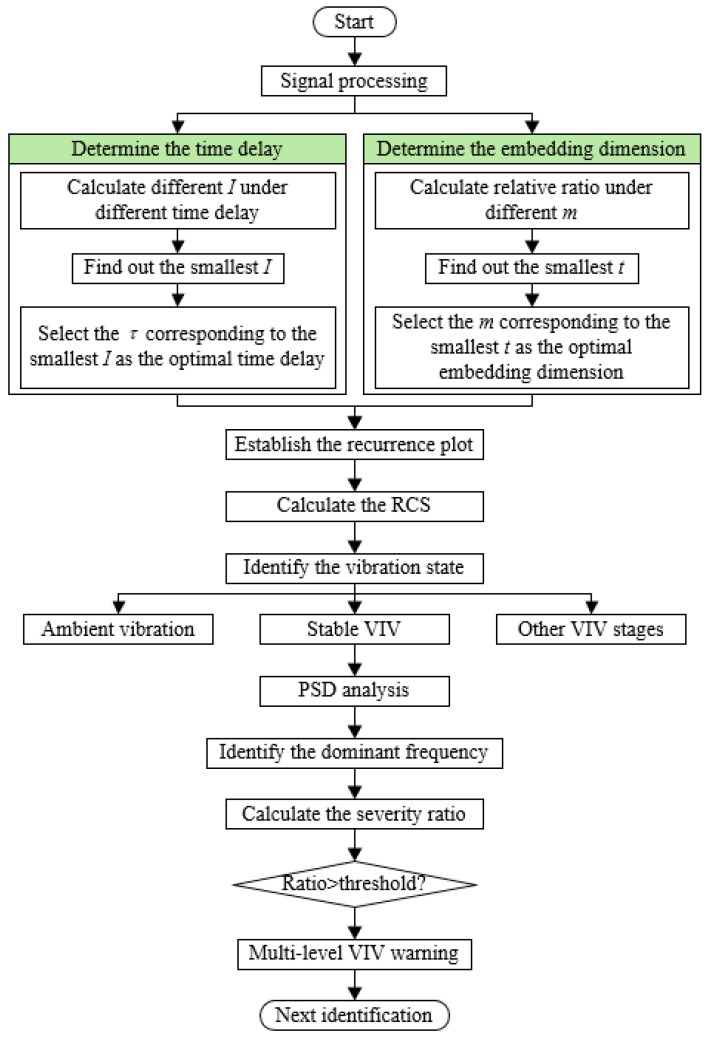
Flowchart of automated VIV identification process.

By introducing the recurrence plot and proposing the feature index, the automatic VIV identification process is established. By proposing the severity ratio to estimate the severity of VIV, the multi-level warning strategy can be established. The whole process is explained in Figure 10. In each identification, a segment of vibration data is first extracted, and the signal is then processed to remove the noise in the signal. Then MI and FNN methods are conducted to calculate the optimal time delay and embedding dimension, and the recurrence plot is established subsequently. The RCS value is then calculated according to Equation (14), and the vibration state is identified. When a VIV is identified, the power spectral density analysis is conducted to extract the dominant frequency of the VIV. Finally, the severity ratio is computed, and the warning is released based on the severity ratio.

## 4. Application for a Suspension Bridge

### 4.1. Description of the Bridge

**Figure 11:**
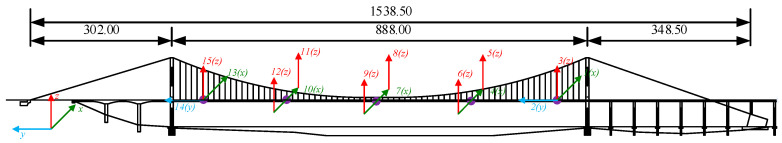
Sensor layout of the bridge.

The prototype bridge located in Guangdong Province in China is a single-span, two-hinged suspension bridge with a span distribution of 302 + 888 + 348.5 = 1538.5 m. The girder of the bridge is a flat, closed, streamlined steel-box girder with a width of 35.6 m and a height of 3 m. A structural health monitoring system was installed on the bridge to monitor the state of the bridge, including 36 channels for vibration monitoring. Eight channels are used to monitor the vibration of the girder with a sampling frequency of 50 Hz. The distribution of the accelerometers on the girder is presented in Figure 11.

**Figure 12:**
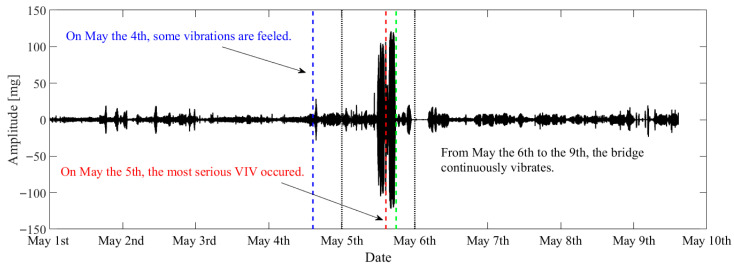
Original data of the bridge during the VIV event.

From the 4th of May to the 6th of May 2020, many VIVs were observed on the bridge. The most serious VIV occurred in the afternoon on the 5th of May and lasted for almost 5 h. The traffic was suspended for driving safety one hour after the obvious VIV was observed. VIV occured multiple times intermittently. The monitoring system recorded the whole process of VIV. To validate the feasibility of the proposed method, 9 days of vibration data from the 1st of May to the 9th of May were utilized for VIV identification. The original data and the VIV events that can be visually observed are highlighted in Figure 12.

### 4.2. Automated VIV Identification Result

The monitoring data from the 8th channel in the middle of the girder is utilized for VIV identification. In each analysis, one minute of monitoring data is used for identification and the continuous identification moves with a step of one minute. Under this parameter setting, every minute of vibration state can be identified and the evolution of the vibration state can be tracked.

**Figure 13:**
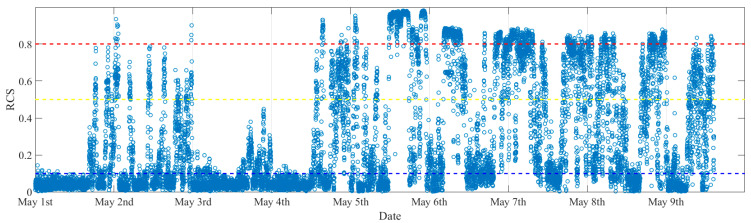
The evolution of RCS.

Figure 13 presents the evolution of the calculated RCSs for each one minute of data, in which the blue-, yellow-, and red-dash lines represent the thresholds for different vibration states. It can be seen that from the 1st of May to the 9th of May, many RCSs were greater than the third level threshold, denoting that many minutes of stable VIVs occurred in this time period.

**Figure 14:**
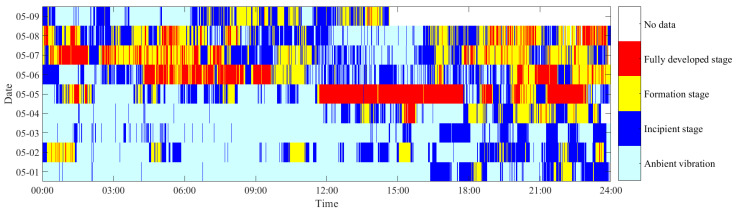
The multi-level identification results of the whole time period.

To more clearly show the evolution of the vibration state, a cloud diagram is presented to show the multi-stage identification results, as shown in Figure 14. It can be seen that continuous VIV occurred from 12:00 to 18:00 on the 5th of May, which matches with the observed vibration. It also can be seen that besides the serious VIV on the 5th of May, many stable VIVs were identified on other days. On the 1st of May, some VIVs in the formation stage are identified, and some discrete, fully developed VIVs are identified on the 2nd of May. The most serious VIV is identified in the afternoon of 5th of May, and this is the VIV observed visually. After the 5th of May, many fully developed VIVs were identified, and these VIVs were not observed and reported because the vibration amplitudes were small.

### 4.3. Validation of Identification Results

**Figure 15:**
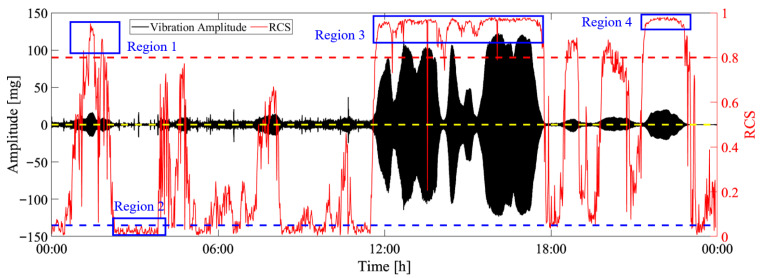
The identification results on the 5th of May.

**Figure 16:**
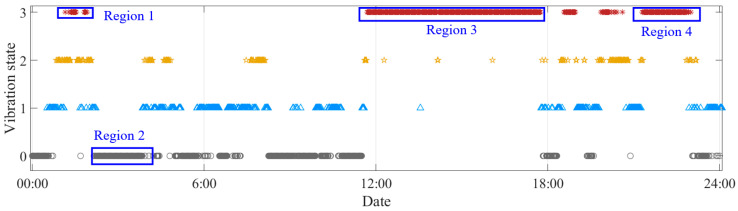
Multi-stage identification results.

To more clearly validate the feasibility of the proposed method, the identification results on 5th of May are analyzed in detail. Figure 15 presents the original data, the calculated RCS and the thresholds. It can be seen that the RCS gradually increases when the vibration amplitude increases and becomes stable, which means the RCS can reflect the vibration state. Four regions are selected to validate the identification results, in which region 1, region 3, and region 4 are identified as stable VIVs and region 2 is identified as ambient vibration. Figure 16 presents the multi-stage identification results, in which 0–3 represent ambient vibration, incipient stage, formation stage, and fully developed stage.

**Figure 17:**
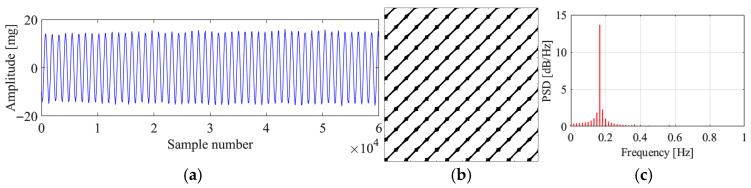
Original data, recurrence plot and PSD plot for region 1. (**a**) Original data; (**b**) recurrence plot; (**c**) PSD plot.

Figure 17 presents the original signal, the corresponding recurrence plot, and the PSD plot for region 1. It can be seen that the original signal is a sine wave signal with stable vibration amplitude around 18 mg. The original signal denotes that the corresponding vibration state is VIV. The recurrence plot shows clear multiple parallel diagonal lines, showing the characteristics of a VIV signal. The PSD plot shows single peak, which means only one mode dominates the vibration, matching with the characteristic that the VIV is a single mode vibration. The proposed method identifies the vibration state as stable VIV, which validates the feasibility of the proposed method in VIV identification.

**Figure 18:**
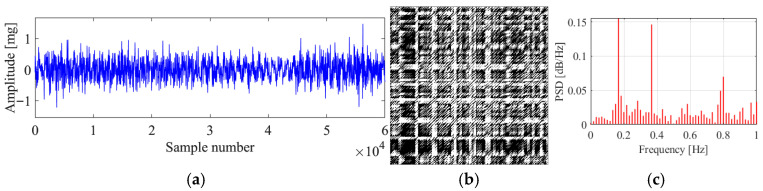
Original data, recurrence plot and PSD plot for region 2. (**a**) Original data; (**b**) recurrence plot; (**c**) PSD plot.

Figure 18 presents the original signal, the corresponding recurrence plot, and the PSD plot for region 2. It can be seen from the vibration signal that the bridge is in an ambient vibration state. The recurrence plot of the signal shows no pattern, and no parallel diagonal lines are formed, which matches with the characteristic of the ambient vibration signal. The PSD plot also shows multiple peaks, which means the vibration is dominated by many modes. The proposed method identifies the vibration state as ambient vibration, validating the correctness of the proposed method.

**Figure 19:**
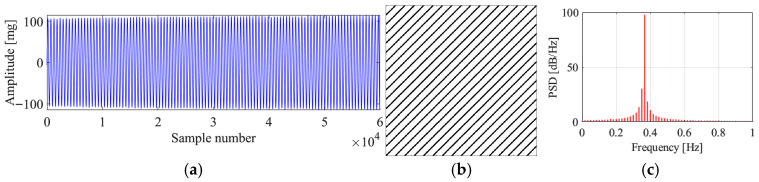
Original data, recurrence plot, and PSD plot for region 3. (**a**) Original data; (**b**) recurrence plot; (**c**) PSD plot.

**Figure 20:**
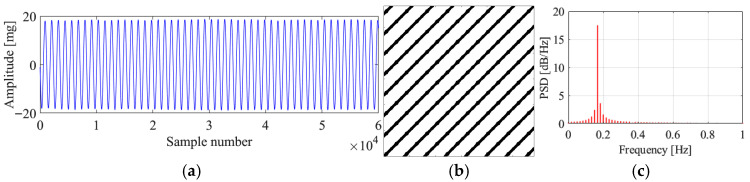
Original data, recurrence plot, and PSD plot for region 4. (**a**) Original data; (**b**) recurrence plot; (**c**) PSD plot.

Figure 19 and Figure 20 present the original signals, the corresponding recurrence plots, and the PSD plots for region 3 and region 4. From the original signals, we can conclude that these two vibrations are stable VIVs. The recurrence plots and PSD plots also show the characteristics of stable VIV. The proposed method successfully identifies these two VIVs, validating the novelty of the proposed method.

One phenomenon needs to be mentioned is that even though the vibration states for regions 1, 3, and 4 are all stable VIVs, the VIV signals are with different amplitudes. The VIV signals for region 1 and 4 are with much smaller vibration amplitude than that for region 3. The proposed method identifies all the VIVs in these three regions, which validates that the proposed method can identify the VIV no matter what amplitude the vibration has. Apart from the amplitude, it can be seen that the dominant frequencies of the VIVs for regions 1, 3, and 4 are different, and the identification results also validate that the proposed method can identify the VIVs with different dominant frequencies. The difference in the dominant frequencies also can be reflected in the recurrence plot. For a small dominant frequency, i.e., big vibration period, a big optimal time delay would be obtained, and sparse diagonals would be observed in the recurrence plot, while the diagonals would be much denser for VIV with bigger dominant frequency. Regardless of whether the recurrence plots have dense or sparse diagonals, the distances between the diagonals stay stable, and this is the reason why the proposed method can successfully identify all the VIVs with different frequencies and vibration amplitudes.

**Figure 21:**
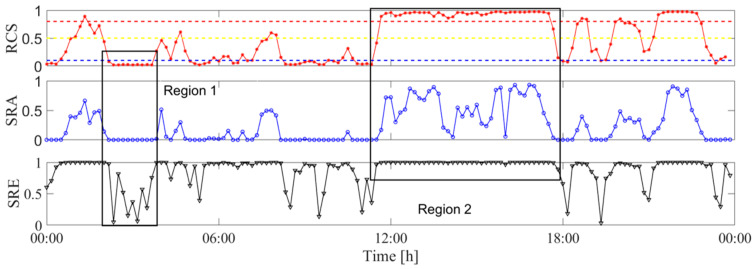
Comparison between the proposed method and the reference method.

To more clearly show the correctness and novelty of the proposed method, a comparison with the existing method is conducted. The reference method [24] utilizes similarity ratio of amplitude (SRA) and similarity ratio of energy (SRE) as the feature index to identify the VIV. Since the reference method utilizes ten minutes of data in each analysis, ten minutes of data are also used in the proposed method. Figure 21 presents the evolution of RCS, SRA, and SRE.

It can be seen that the trend of RCS is generally consistent with that of SRA and SRE, indicating that the proposed method almost produces the same identification method as the reference method. Region 1 shows the values of the three indices. It can be seen that RCS has the same trend as SRA, indicating that the proposed index and the reference index both can estimate the stability of the signal. The vibration states in region 1 are identified as ambient vibrations, and the results also match with the real vibration state. Region 2 shows the values of the three indices where the vibration states in this region are identified as VIVs. The SREs are close to 1, indicating that the vibration in this region is single-mode dominated. Most of the SRAs are close to 1, and this indicates that the signals in this region are with stable vibration amplitude. Even though the identification results are the same for the proposed and the reference method, RCS is more stable than SRA, indicating that the proposed method can produce stable VIV identification results.

A difference should be noted that the reference method only produces values of SRE and SRA. The change in these indices are not friendly for engineers to confirm the identification results. The proposed method can visualize the characteristics of the signal (see Figure 17, Figure 18, Figure 19 and Figure 20) and provides an intuitive tool to confirm the identification results.

### 4.4. Validation of Multi-Level VIV Warning

**Figure 22:**
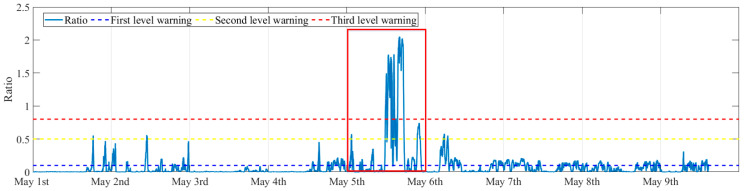
The evolution of severity ratio.

Figure 22 presents the evolution of the severity ratio during the 9 days. It can be seen that the ratios are smaller than the second-level threshold for most of the time, and it reaches the third-level warning on the 5th of May, which means the VIV amplitude exceeds the allowable displacement amplitude.

**Figure 23:**
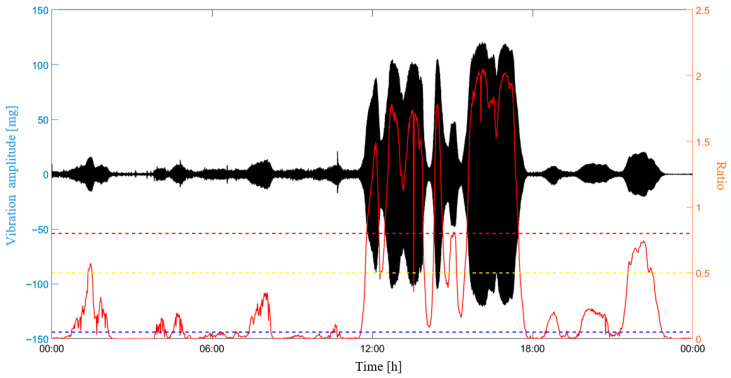
The evolution of severity ratio on the 5th of May.

Figure 23 presents in detail the evolution of the severity ratio with the vibration on the 5th of May, which is highlighted in Figure 22 with a red box. It can be observed that the severity ratio increases when VIV is identified with a large vibration amplitude. The most serious VIV occurs from 12:00 to 18:00. During this time period, the severity ratio exceeds the threshold of the third-level warning. The VIV warning matches with the reality that the most obvious VIV was observed during this time period, indicating the rationality of the proposed warning index and warning strategy.

It is worth noting that a second-level warning is issued at about 1:00 on this day when the VIV is identified. This warning actually can give an early warning to the bridge’s engineering management that more serious VIVs would probably occur in the future if the wind does not change. For a VIV with large vibration amplitude, an earlier small-amplitude VIV is a necessary precursor, because the vibration process of a small-amplitude VIV accumulates vibration energy. When a small amplitude VIV occurs and a stable wind continues, a more serious VIV is very likely to occur in the future. The multi-level warning system provides alerts for early VIVs; therefore, it can provide early warning for a possible large-amplitude VIV.

### 4.5. Parametric Analysis

**Figure 24:**
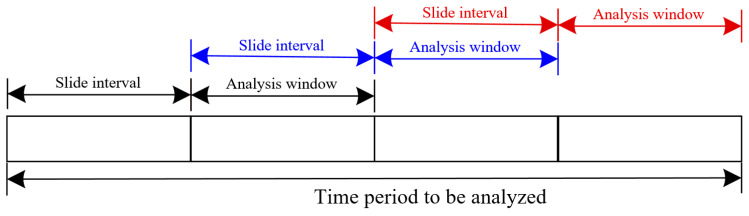
Principle of analysis window and slide interval.

In real applications, the VIV identification is expected to be operated online and in a continuous way to fulfill the online continuous identification and warning system. The moving window strategy is the optimal way to fulfill the online identification. In the moving window strategy, the analysis window and the slide interval are two parameters that need to be carefully set. The analysis window means the time period of the data to be analyzed and the slide interval means the time period of the data moves forward after each analysis. The principle of analysis window and slide interval is presented in Figure 24. It can be seen from the figure that the slide interval is suggested to be set the same as the analysis window. Under these circumstances, all the collected data would be analyzed without any overlapping.

The analysis window determines the number of data to be analyzed in each identification. When a big analysis window is used, i.e., more data are utilized in one identification, it would cause more time in the process of signal processing and result visualization. In continuous identification, computing time is required to be smaller than the analytical window to avoid time delays.

Table 4 presents the computing time consumed in the identification process when a different analysis window is set, in which computing time 1 denotes the time consumed without the process of visualizing the identification results and computing time 2 denotes the time including the process visualizing the recurrence plot.

It can be seen that the computing time increases when the analytical window is set bigger, and the computing time increases nonlinearly when the analytical window increases. It also can be seen that Computing Time 2 is much bigger than Computing Time 1, indicating that visualizing the identification results costs much more time than the other process. When the Analytical Window is set up for longer than 3 min, Computing Time 2 reaches 338 s, which exceeds the Analytical Window and would lead to a time delay in continuous identification. Therefore, a small analytical window is suggested in practical application.

In practical applications, the analytical window is suggested to be set as 1 min. For the monitored VIV occurring at the girder, the formation stage and fully developed stage of VIV both last for tens of minutes. One minute of monitoring data can correctly reflect the current vibration state and guarantee a correct identification result. In addition, one minute of monitoring data would not cause too much computing time and the identification process can output visualized identification results for verification of VIV for engineers.

## 5. Conclusions

In this study, a novel VIV identification and warning method is proposed. A recurrence plot is introduced to graph the vibration signal. A feature index defined as RCS is proposed to quantify the regularity and stability of the signal, and different vibration states can be identified according to the RCS. Multi-level warning is also established based on the vibration amplitude of the VIV. The optimal characteristic parameters of the recurrence plot, i.e., the time delay and the embedding dimension, are automatically determined using MI and FNN methods. An automatic VIV identification and warning process is established, and the feasibility is validated through a real application to a suspension bridge. The conclusions can be drawn as follows:

(1) The recurrence plot can visualize the difference in the signals corresponding to different vibration states, and it shows multiple parallel diagonal lines for VIV signals while it shows no specific pattern for ambient vibration. The recurrence plot has the advantage in graphing the characteristic of the signal and is very suitable for online identification.
(2) The proposed RCS can estimate the stability of the diagonal lines (vibration signal), and therefore it is capable of identifying the vibration state. For VIV signal, the recurrence plot would show stable parallel diagonal lines and have small RCS, while the RCS would be big for ambient vibration.
(3) The proposed method is capable of identifying the VIVs with small and big vibration amplitudes and different dominant modes. The recurrence plot shows the same characteristics for VIVs with large and small vibration amplitudes. Since the proposed RCS estimates the stability of the signal without any limitation on vibration amplitude, it can be used for identifying VIVs with any amplitude.
(4) The proposed severity ratio can estimate the severity of the VIV through comparing the vibration amplitude with the allowable displacement amplitude. The multi-level warning can successfully warn the VIV with different severities and provide possible early warning for serious VIV.
(5) The processes of determining the optimal parameters, building the recurrence plot, calculating the RCS, identifying the vibration state, and issuing the warning are fully automated. The proposed method can be used for automated VIV identification and continuous online VIV identification without any manual intervention.
(6) One of the novelties of the proposed method is the visualization of the identification results. If a large analytical window is utilized, the visualization procedure would cost too much time, leading to an efficiency problem in real time identification. In real application, the analytical window is suggested to set to 1 min to guarantee a precise identification result and an efficient real time analysis.

## Figures and Tables

**Table 1:** Characteristics of signals corresponding to different vibration states.

Vibration State	Original Signal	Recurrence Plot	Statistic of Vertical Distance	RCS
Ambient vibration	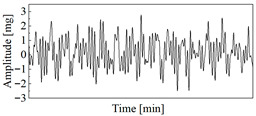	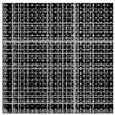	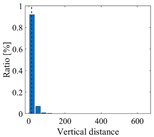	0.023
Formation stage of VIV	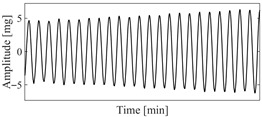	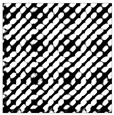	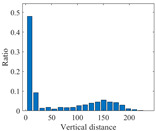	0.254
Stabilized stage of VIV	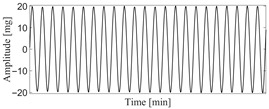	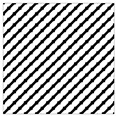	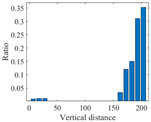	0.895

**Table 2:** Thresholds for multi-stage VIV identification.

Vibration State	Ambient Vibration	Incipient Stage	Formation Stage	Fully Developed Stage
RCS	0.0–0.1	0.1–0.5	0.5–0.8	0.8–1.0

**Table 3:** Multi-level warning strategy and thresholds.

Warning Level	Threshold
No warning	(0, 0.05]
First level warning	(0.05, 0.5]
Second level warning	(0.5, 0.8]
Third level warning	(0.8~)

**Table 4:** Computing time under different analytical times.

Analytical Window (Unit: min)	Computing Time 1 (Unit: s)	Computing Time 2 (Unit: s)
1	1.7	10.3
2	6.9	61
3	16.4	338
4	37.5	473

## Data Availability

Some or all data, models, or code that support the findings of this study are available from the corresponding author upon reasonable request.
